# Full-length genome sequence of segmented RNA virus from ticks was obtained using small RNA sequencing data

**DOI:** 10.1186/s12864-020-07060-5

**Published:** 2020-09-16

**Authors:** Xiaofeng Xu, Jinlong Bei, Yibo Xuan, Jiayuan Chen, Defu Chen, Stephen C. Barker, Samuel Kelava, Xiaoai Zhang, Shan Gao, Ze Chen

**Affiliations:** 1 Hebei Key Laboratory of Animal Physiology, Biochemistry and Molecular Biology, College of Life Sciences, Hebei Normal University, Shijiazhuang, Hebei 050024 People’s Republic of China; 2 Guangdong Provincial Key Laboratory for Crop Germplasm Resources Preservation and Utilization, Agro-Biological Gene Research Center, Guangdong Academy of Agricultural Sciences, Guangzhou, Guangdong 510640 People’s Republic of China; 3 Guangdong Laboratory for Lingnan Modern Agriculture, Guangzhou, Guangdong 510642 People’s Republic of China; 4 College of Life Sciences, Nankai University, Tianjin Tianjin, 300071 People’s Republic of China; 5 School of Chemistry and Molecular Biosciences, The University of Queensland, Brisbane, QLD 4072 Australia

**Keywords:** MGTV, JMTV, Full-length genome, 5′ sRNA, 3′ sRNA

## Abstract

**Background:**

In 2014, a novel tick-borne virus of the *Flaviviridae* family was first reported in the Mogiana region of Brazil and named the Mogiana tick virus (MGTV). Thereafter, the Jingmen tick virus (JMTV), Kindia tick virus (KITV), and Guangxi tick virus (GXTV)—evolutionarily related to MGTV—were reported.

**Results:**

In the present study, we used small RNA sequencing (sRNA-seq) to detect viruses in ticks and discovered a new MGTV strain in *Amblyomma testudinarium* ticks collected in China’s Yunnan Province in 2016. We obtained the full-length genome sequence of this MGTV strain Yunnan2016 (GenBank: MT080097, MT080098, MT080099 and MT080100) and recommended it for its inclusion in the NCBI RefSeq database for future studies on MGTV, JMTV, KITV and GXTV. Phylogenetic analysis showed that MGTV, JMTV, KITV and GXTV are monophyletic and belong to a MGTV group. Furthermore, this MGTV group of viruses may be phylogenetically related to geographical regions that were formerly part of the supercontinents Gondwana and Laurasia.

**Conclusions:**

To the best of our knowledge, this is the first study in which 5′ and 3′ sRNAs were used to generate full-length genome sequences of, but not limited to, RNA viruses. We also demonstrated the feasibility of using the sRNA-seq based method for the detection of viruses in pooled two and even possible one small ticks. MGTV may preserve the characteristic of ancient RNA viruses, which can be used to study the origin and evolution of RNA viruses. In addition, MGTV can be used as novel species for studies in phylogeography.

## Background

Next generation sequencing (NGS) technologies have been widely applied to virus and viroid discovery in plants and animals. Compared to other NGS based methods, the small RNA sequencing (sRNA-seq) based method simplifies virus detection and has several other advantages [1]. The sRNA-seq based method was originally used for viral detection and identification in plants [2] and in invertebrates [3]. Although the sRNA-seq based method does not perform as well in the detection of mammalian viruses as in the detection of plant or invertebrate viruses, we still detected eight mammalian viruses: human papillomavirus type 18 (HPV-18) [4], hepatitis B virus (HBV) [4], hepatitis C virus (HCV) [4], human immunodeficiency virus type 1 (HIV-1) [4], squirrel monkey retrovirus (SMRV) [4], Epstein-Barr virus (EBV) [4], severe acute respiratory syndrome coronavirus (SARS-CoV) [5] and a DNA segment of African swine fever virus (ASFV) [6]. The discovery of featured RNA fragments including small interfering RNA (siRNA) duplexes [7], 5′ and 3′ end sRNAs [8, 9] palindromic sRNAs (psRNAs) and complemented psRNAs (cpsRNAs) [5] increased our capacity to detect viruses in mammals. Moreover, we found that 5′ and 3′ sRNAs can be used to annotate nuclear non-coding and mitochondrial genes at 1 bp resolution [10, 11] In the present study, we report that 5′ and 3′ sRNAs can be used to complete 5′ and 3′ ends of genome sequences of RNA viruses at 1-bp resolution and generate full-length genome sequences.

Ticks transmit a multitude of infectious agents to humans and other animal species, including viruses of the *Flaviviridae* family, which are among the most common tick-borne viruses [12]. With the widespread use of NGS, a number of studies have applied metagenomic methods to detect tick-associated pathogens [13]. However, metagenomic methods using DNA cannot be used to detect RNA viruses without DNA stages. Consequently, transcriptomic approaches using total RNA have also been used to detect tick viruses [14]. The sRNA-seq based method has been successfully used in the detection of *Rickettsia* in ticks [15]. To the best of our knowledge, there are no previous studies of virus detection in ticks using the sRNA-seq based method until the detection of the DNA segment of ASFV [6]. Although high-depth sRNA-seq data was used to detect a DNA segment of ASFV, the coverage of the ASFV reference genome was still very low. This suggested that the sRNA-seq based method does not perform as well in the detection of DNA viruses as it does in the detection of RNA viruses.

In a previous study [6], we used sRNA-seq to detect viruses in ticks. Subsequent analysis of these viruses led to the discovery of a new strain of RNA virus. In 2014, the virus was first reported as a novel tick-borne virus of the *Flaviviridae* family in the Mogiana Region of Brazil [12] and was named Mogiana tick virus (MGTV). Subsequently, Jingmen tick virus (JMTV), Kindia tick virus (KITV) and Guangxi tick virus (GXTV)—evolutionarily related to MGTV (Results and Discussion)—were detected in ticks. In 2018, viruses closely related to JMTV were detected in the sera samples of three Crimean-Congo hemorrhagic fever (CCHF) patients collected from 2013 and 2015 in Kosovo and two of these patients had exposed to tick bites [16]. In the present study, we identified a new MGTV strain Yunnan2016 detected in *Amblyomma testudinarium* ticks [17] and aimed to achieve the following research goals: (1) establish a method to generate the full-length genome sequence of an RNA virus using sRNA-seq data; (2) determine the feasibility of using the sRNA-seq based method in the detection of viruses in a small tick; and (3) provide a high-quality and well-curated reference genome for future studies on MGTV, JMTV, KITV and GXTV.

## Results and discussion

### Detection of viruses in ticks using sRNA-seq data

**Fig. 1:**
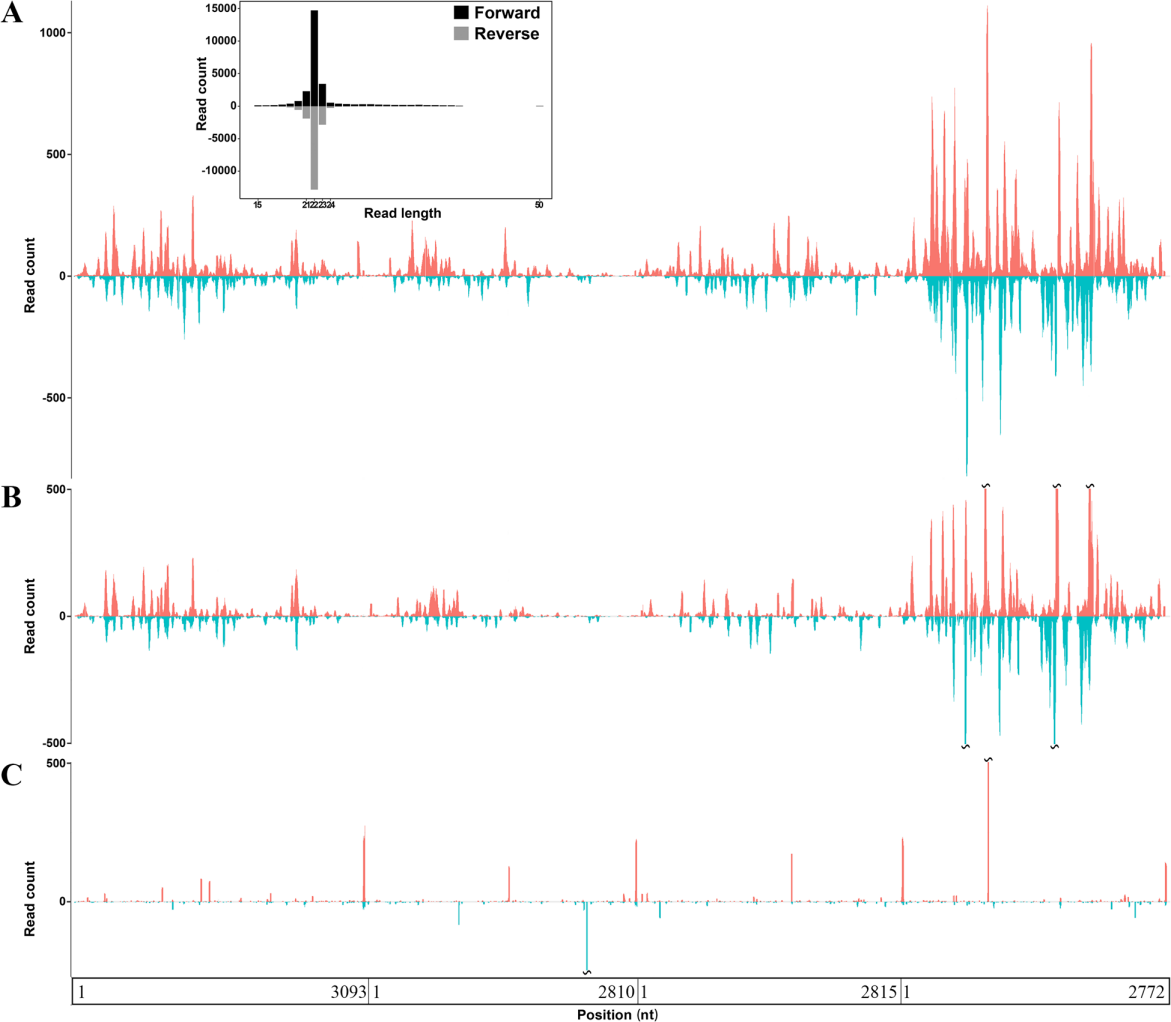
Genome coverage and average depth of the MGTV strain Yunnan2016. The y-axis represents the read-counts for each genomic position. **a.** The x-axis represents positions in the reference genome of the MGTV strain Yunnan2016 (GenBank: MT080097, MT080098, MT080099 and MT080100) and the sRNA-seq data SRR4116826 (Table 1) was aligned to this reference genome; **b.** The x-axis represents positions on the reference genome of the MGTV strain Xinjiang2016 (GenBank: MK174251, MK174244, MK174230 and MK174237) and the sRNA-seq data SRR4116826 (Table 1) was aligned to this reference genome; **c.** The x-axis represents positions on the reference genome of the MGTV strain Yunnan2016 and the sRNA-seq data SRR8439389, SRR8439390, SRR8432408, SRR8432409, SRR811197093 and SRR811197094 (Table 1) were aligned to this reference genome as negative controls

*Amblyomma testudinarium, Dermacentor nuttalli, D. niveus* and *D. silvarum* ticks were collected for our previous studies [8, 18]. The sRNA-seq data from these ticks were generated and deposited in the NCBI SRA database under the accession numbers SRP084097 and SRP178347 (Table 1). Using VirusDetect (Refer to Methods), MGTV was detected in *A. testudinarium* ticks (SRA: SRP084097) but not in *D. nuttalli, D. niveus* or *D. silvarum* (SRA: SRP178347) ticks that were used as negative controls. Since the *A. testudinarium* ticks were collected in China’s Yunnan Province in 2016, the new MGTV strain was named Yunnan2016. As a segmented RNA virus, MGTV is composed of four RNAs in its genome, two of which (RNA1 and RNA3) are related to the nonstructural protein genes of the genus *Flavivirus* (family *Flaviviridae*), while the other two segments (RNA2 and RNA4) are unique to MGTV. VirusDetect (Refer to Methods) uses the closest reference sequence to report the detected virus. Used as reference to report Yunnan2016, the genome of the JMTV strain Xinjiang2016 (GenBank: MK174251, MK174244, MK174230 and MK174237) was sequenced from wild rodents collected in China’s Xinjiang Province. The sRNA-seq data from *A. testudinarium* (SRA: SRR4116826) covered 86.71% of the Xinjiang2016 genome with an average depth of 46.66 (Table 1). The sRNA-seq data from the *A. testudinarium* contained significantly more reads aligned to the Yunnan2016 genome (Fig. 1a) and the Xinjiang2016 genome (Fig. 1b) than the sRNA-seq data from the three other species (Fig. 1c). RNA1, RNA2, RNA3 and RNA4 of the MGTV strain Yunnan2016 were assembled at the contig level. Subsequently, PCR amplification coupled with Sanger sequencing (Additional file 1) was used to fill the gaps between contigs and confirm the genome assembly: 93.7% (2879/3073) of RNA1, 90.6% (2528/2790) of RNA2, 88.3% (2468/2795) of RNA3 and 95.2% (2619/2752) of RNA4 were confirmed by Sanger sequencing [the polyA tails of 3′ untranslated regions (UTRs) were not part of these calculations].

**Table 1:** Genome coverage and average depth of the MGTV strain Yunnan2016

Data	Tick	Reference	Coverage	Depth
SRR4116826	*Amblyomma.* *testudinarium*	Xinjiang2016	86.47%	46.52
		Yunnan2016	97.37%	91.11
SRR8439389 SRR8439390	*Dermacentor.* *nuttalli*	Yunnan2016	18.40%	2.58
SRR8432408	*D. niveus*	Yunnan2016	18.62%	2.91
SRR8432409		Yunnan2016	20.72%	2.94
SRR811197093	*D. silvarum*	Yunnan2016	24.38%	10.31
SRR811197094		Yunnan2016	8.11%	4.22

“Data” (the first column) was aligned to the reference genome (the 3rd column) to obtain the information on the fourth and fifth columns. “Data” was unique for each run of sRNA-seq data in the NCBI SRA database. The virus strain Yunnan2016 (GenBank: MT080097, MT080098, MT080099 and MT080100) was detected in the present study. The reference genome of the MGTV strain Xinjiang2016 (GenBank: MK174251, MK174244, MK174230 and MK174237) was used to report Yunnan2016. “Coverage” and “Depth” indicated the genome coverage and average depth, respectively (Refer to Methods)

### Full-length genome sequence of the MGTV strain Yunnan2016

**Fig. 2:**
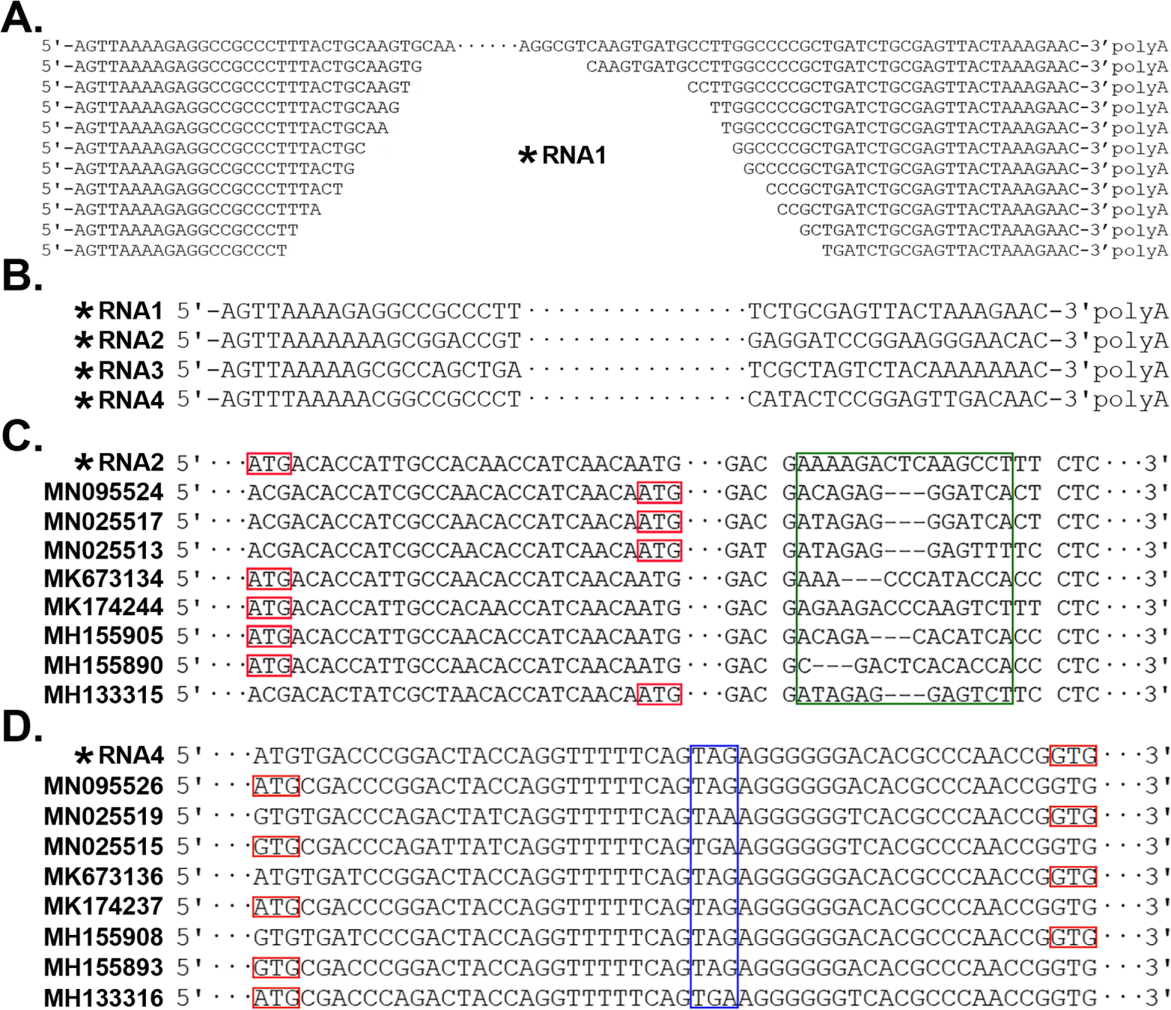
The full-length genome sequence of the MGTV strain Yunnan2016. * RNA1, RNA2, RNA3 and RNA4 of the MGTV strain Yunnan2016 were submitted to the GenBank under the accession numbers MT080097, MT080098, MT080099 and MT080100, respectively. **a.** 5′ and 3′ end sRNAs were used to generate the full-length 5′ and 3′ ends of RNA1; **b.** 5′ and 3′ ends of all RNAs in the Yunnan2016 genome; **c.** Start codons are marked in red boxes and RNA2 has a 15- or 18-nt variable region; **d.** Start codons and stop codons are marked in red and blue boxes, respectively. For RNA4 of some viruses, “GTG” were identified as the start codons of the second ORF and the nearby “ATG” codons (not shown in this figure) are 48 nt downstream of the “GTG” codons

We used 5′ and 3′ sRNAs (Fig. 2a) to generate the full-length genome sequence of the new MGTV strain Yunnan2016 at 1 bp resolution (Additional file 1). The 5′ ends of all RNAs in the Yunnan2016 genome have the sequence motif AG [T]_2–3_[A]_4–6_[C/G]_n_AAGTGC (Fig. 2b), where [C/G]_n_ represents a GC-enriched region. The 3′ ends of all RNAs in the Yunnan2016 genome have an AC-enriched region (Fig. 2b). RNA1, RNA2, RNA3 and RNA4 of Yunnan2016 with respective lengths of 3093, 2810, 2815 and 2772 nt were submitted to the NCBI GenBank database under the accession numbers MT080097, MT080098, MT080099 and MT080100, respectively. The length of the polyA tail in each 3′-UTR of these RNAs was set as 20 nt. The sRNA-seq data from *A. testudinarium* (SRA: SRR039620) covered 97.37% of the full-length genome sequence of Yunnan2016 with an average depth of 91.11 (Table 1); 58.5% (26,668/45,563) of the virus reads were aligned to RNA4 (Fig. 1b). Although MGTV is a positive-sense single-stranded RNA (+ssRNA) virus, the proportion of sRNA-seq reads aligned to the viral positive- and negative-strands was 1.42 (26,767/18,796).

Compared to the genome coverage of 86.47% and average depth of 46.52 (Refer to Methods) when we used the Xinjiang2016 genome as a reference (Fig. 1a), genome coverage increased to 97.37% and average depth to 91.11 when we used the Yunnan2016 genome as a reference (Fig. 1b). A high-quality virus genome should contain the full-length 5′ UTRs, as these regions contain useful information for the analysis of such genomes. In a previous study, we analyzed 5′ UTRs in Betacoronaviruses and developed 5′-UTR barcoding to be used in detection, identification, classification and phylogenetic analysis of, but not limited to, coronaviruses [19]. Comparing the full-length genome sequence of Yunnan2016 with those of 16 other MGTV, JMTV, KITV and GXTV complete genomes (Refer to Methods), we found that none of these other genomes had the correct full-length 5′ and 3′ ends. Particularly, RNA1 (GenBank: MN025516) and RNA4 (GenBank: MN025515) of the JMTV strain TT2017–2 had 56 and 48 nt additional sequences at their 5′ ends, respectively. Further analysis showed the additional sequences were identical to internal regions of the genomes. Obviously, these additional sequences had been assembled incorrectly in the previous studies. Therefore, this high-quality and full-length genome sequence of the MGTV strain Yunnan2016 should be included in the NCBI RefSeq database for future studies on MGTV, JMTV, KITV and GXTV.

### Phylogenetic analysis of MGTV genomes

**Fig. 3:**
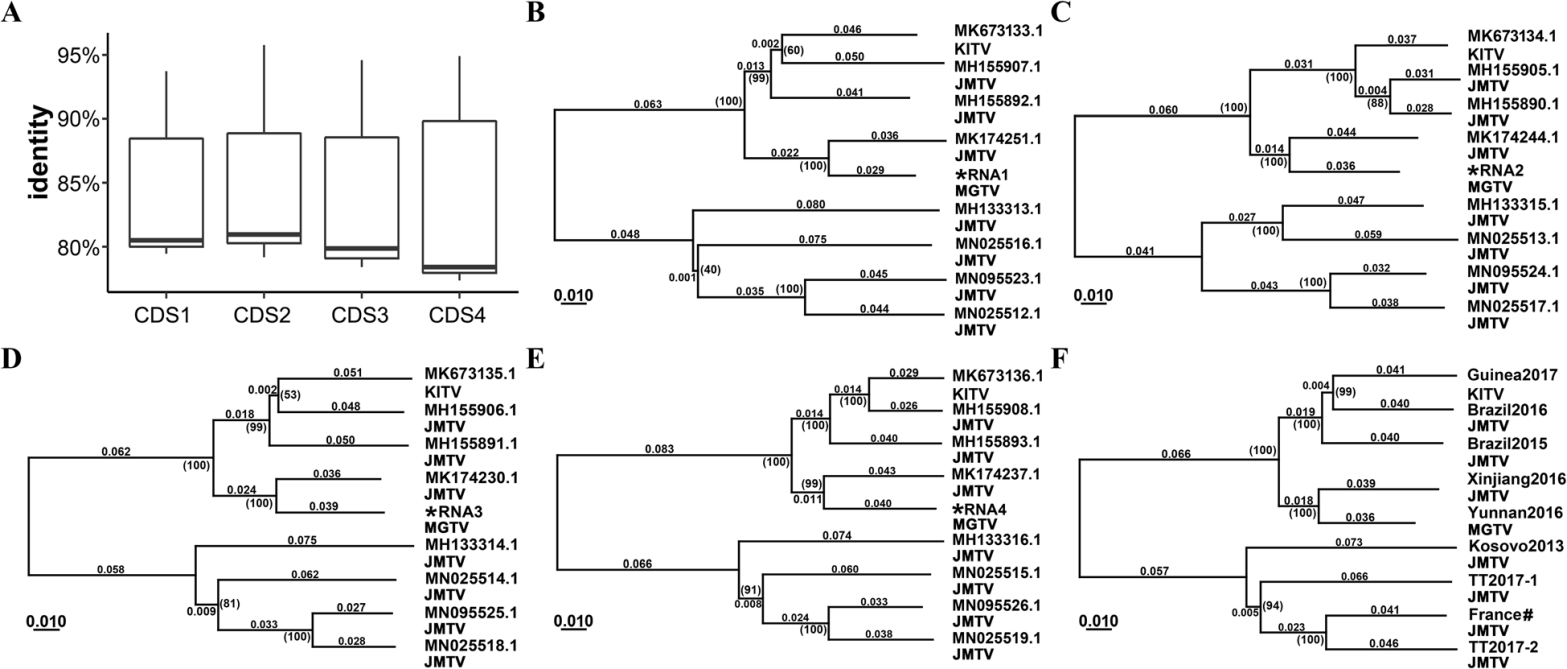
Phylogenetic analysis of MGTV, JMTV, KITV and GXTV. * RNA1, RNA2, RNA3 and RNA4 of the MGTV strain Yunnan2016 were submitted to the GenBank under the accession numbers MT080097, MT080098, MT080099 and MT080100, respectively. The first reported MGTV strain (JX390986.2, KY523073.1, JX390985.2 and KY523074.1) and the only GXTV strain (MG703253.1, MG703254.1, MG703252.1 and MG703255.1) with the identities of 93.85 and 94% to the strains Guinea2017 and Yunnan2016, respectively, were removed as redundant sequences. **a.** Paired Pearson correlations between CDSs of 17 viruses were used to account for the degrees of evolutionary conservation among four CDSs. Five phylogenetic trees were built from the 2745-nt (**b**), 2265-nt (**c**), 2427-nt (**d**), 2351-nt (**e**) and the combined 9788-nt CDSs (**f**) using Unweighted Pair Group Method with Arithmetic Mean (UPGMA), Maximum Parsimony (MP) and Neighbor Joining (NJ) methods. Here, we only show the result using the NJ method with a bootstrap test (1000 replicates). The bootstrap values (marked by parentheses) were in the format for displaying percentages with “%” omitted. The virus strains were collected from Martinique of France# (Central America), Trinidad and Tobago (Central America), Kosovo (Central Europe), Brazil (South America), Guinea (West Africa), Xinjiang (Northeast of China) and Yunnan (Southeast of China).TT: Trinidad and Tobago

In total, 17 MGTV, JMTV, KITV and GXTV genomes were used for the further analysis (Refer to Methods). Five protein-coding genes were annotated for each of 17 genomes. The putative proteins encoded by RNA1, RNA2 and RNA3 are the RNA-dependent RNA polymerase, glycoprotein and protease, respectively, whereas the putative proteins encoded by RNA4 are the capsid protein and the membrane protein. The RNA-dependent RNA polymerase from RNA1, the protease from RNA3, and the capsid protein from RNA4 had lengths of 915, 809 and 255 aa (amino acid residues), respectively. In all 17 virus genomes, the lengths of these three proteins were constant, whereas those of the other two proteins (the glycoprotein from RNA2 and the membrane protein from RNA4) varied. The lengths of the glycoprotein from RNA2 varied because of two mutations (Fig. 2c): T/C mutations in the start codons shortened the coding sequences (CDSs) of RNA2 by 21 nt and a small insertion/deletion (InDels) shortened the CDSs by 3 nt. Theoretically, four types of glycoproteins with lengths of 745, 746, 754 or 755 aa would be translated from RNA2; however, a glycoprotein with 746 aa was not observed in the 17 virus genomes. Since the lengths of the membrane protein from RNA4 varied because of one mutation—T/C (Fig. 2d)—two types of membrane proteins with lengths of 522 or 539 aa can be translated from RNA4. The multiple-aligned RNA1, RNA3 and RNA4 had 2745, 2427 and 2351 nt CDSs, whereas the multiple-aligned RNA2 had a 2265 nt CDS with a 15 or 18 nt variable region removed (Fig. 2c). CDS 1, 2, 3 and 4 of RNA 1, 2, 3 and 4 could then be connected into a combined 9788 nt CDS. Using paired Pearson correlations between the CDSs of 17 viruses, the degrees of evolutionary conservation are ranked as CDS 2, 1, 3 and 4 (Fig. 3a).

Five phylogenetic trees from the CDS 1, 2, 3 and 4, as well as the combined CDSs, were built using nine non-redundant genome sequences (Refer to Methods). Although CDS 1, 2, 3 and 4 exhibited substantial differences in their degrees of evolutionary conservation, the tree topologies from them remained congruent using the unweighted pair group method with arithmetic mean (UPGMA), maximum parsimony (MP) and neighbour joining (NJ) methods (Fig. 3b-f). MGTV, JMTV, KITV and GXTV belong to an MGTV group with two major clades. The two branches of Clade I contain the virus strains isolated from Brazil (South America) and Guinea (West Africa), in addition to the virus strains Yunnan2016 and Xinjiang2016 (Fig. 3f). Clade II contains the virus strains isolated from Martinique of France (Central America), Trinidad and Tobago (Central America) and Kosovo (Central Europe). Phylogenetic analysis of these viruses in relation to their geographic information showed that Clade I and Clade II approximately correspond with the supercontinents Gondwanaland and Laurasia, respectively. Brazil and Guinea were very close in Gondwanaland, while Martinique of France, Trinidad and Tobago (TT) and Kosovo were close in Laurasia. Based on Wegener’s concept, Pangea is considered to have formed from the assembly of Earth’s continents in the time range of 300–250 Ma (mega-annum: one million years) and consisted of Gondwana (Australia, India, Sri Lanka, Madagascar, East Antarctica, South America and Africa) as its southern half and Laurasia (North America, Greenland, and Eurasia) as its northern half [20]. Our results suggest that the MGTV group of viruses are phylogenetically related to geographical regions that were formerly part of Gondwana and Laurasia.

## Conclusions

In the present study, we conclude: (1) the high-quality, well-curated and full-length Yunnan2016 genome (MT080097, MT080098, MT080099 and MT080100) should be included in the NCBI RefSeq database for future studies on MGTV, JMTV, KITV and GXTV; (2) To the best of our knowledge, this is the first study in which 5′ and 3′ sRNAs were used to generate full-length genome sequences of, but not limited to, RNA viruses; (3) it is feasible to use the sRNA-seq based method for the detection of viruses in pooled two and even possible one small ticks; (4) MGTV may preserve the characteristic of ancient RNA viruses, which can be used to study the origin and evolution of RNA viruses; and (5) MGTV can be used as novel species for studies in phylogeography. Future studies should be conducted to confirm the viability of MGTV in ticks and the hosts of these ticks.

## Methods

The workflow to generate a full-length genome using 5′ and 3′ sRNAs can be seen in the Additional file 1. The full-length genome sequence of the MGTV strain Yunnan2016 has been deposited into NCBI GenBank database under the accession numbers MT080097, MT080098, MT080099 and MT080100. The sRNA-seq data was deposited in the NCBI SRA database under the accession numbers SRP084097 and SRP178347 (Table 1). In the present study, 17 MGTV, JMTV, KITV and GXTV complete genome sequences (including the Yunnan2016) were downloaded from the NCBI GenBank database (Additional file 1) and analyzed together. One genome sequence (GenBank: MN095531, MN095532, MN095533 and MN095534) was removed because it had too many ambiguous nucleotides. The online tool CD-HIT [21] (Date: 20191212) was then used to remove redundant sequences with the sequence identity cut-off 0.93 and default settings for other parameters, resulting in 9 complete genome sequences for the phylogenetic analysis. The multi-alignment of sequences and the phylogenetic analysis were performed using the online tool ClustalW2 [22] and the software MEGA v7.0.26 [23], respectively.

The software Fastq_clean [24] was used for sRNA data cleaning and quality control. The virus detection was performed using the pipeline VirusDetect [25]. For each detected virus, VirusDetect assigned a closest reference genome from the NCBI Genbank database to help characterize that virus. VirusDetect used reference genome coverage and average depth to quantify the detected viruses for validation. Genome coverage was defined as the proportion of read-covered positions divided by genome length whereas average depth was the total number of base pairs of aligned reads divided by the read-covered positions of the reference genome. Statistical computation and plotting were performed using the software R v2.15.3 with the Bioconductor packages [26].

## Supplementary information

**Additional file 1 : Figure S1**. A workflow to generate full-length genome sequence of an RNA virus. Table S1. Collection of ticks. **Table S2**. Primers for PCR amplification coupled with Sanger sequencing. **Table S3**. PCR reagent for each sample. **Table S4**. 17 complete genomes for further analysis.

## Data Availability

The complete genome sequence of Yunnan2016 is available at the NCBI GenBank database under the accession numbers MT080097, MT080098, MT080099 and MT080100. The sRNA-seq data used for virus detection is available at the NCBI SRA database under the accession numbers SRP084097 and SRP178347.

## Abbreviations

NGS: Next generation sequencing
MGTV: Mogiana tick virus
JMTV: Jingmen tick virus
KITV: Kindia tick virus
GXTV: Guangxi tick virus
sRNA-seq: small RNA sequencing
HPV-18: Human papillomavirus type 18
HBV: Hepatitis B Virus
HCV: Hepatitis C Virus
HIV-1: Human immunodeficiency virus type 1
SMRV: Squirrel monkey retrovirus
EBV: Epstein-barr virus
SARS-CoV: Severe Acute Respiratory Syndrome Coronavirus
ASFV: African Swine Fever Virus
cpsRNAs: complemented palindromic small RNAs
CDSs: Coding sequences
InDels: Insertions/Deletions
