# Supplementary material for: Full-length genome sequence of segmented RNA virus from ticks was obtained using small RNA sequencing data
Source: BMC Genomics. 2020 Sep 16;21:641. doi: 10.1186/s12864-020-07060-5 (PMC7493057; doi:10.1186/s12864-020-07060-5)
Supplement: Supplementary file 1 — Additional file 1 : Figure S1. A workflow to generate full-length genome sequence of an RNA virus. Table S1. Collection of ticks. Table S2. Primers for PCR amplification coupled with Sanger sequencing. Table S3. PCR reagent for each sample. Table S4. 17 complete genomes for further analysis. [file 12864_2020_7060_MOESM1_ESM.doc]

# Additional file 1

### **1.1 Full-length genome sequence of the MGTV strain Yunnan2016**


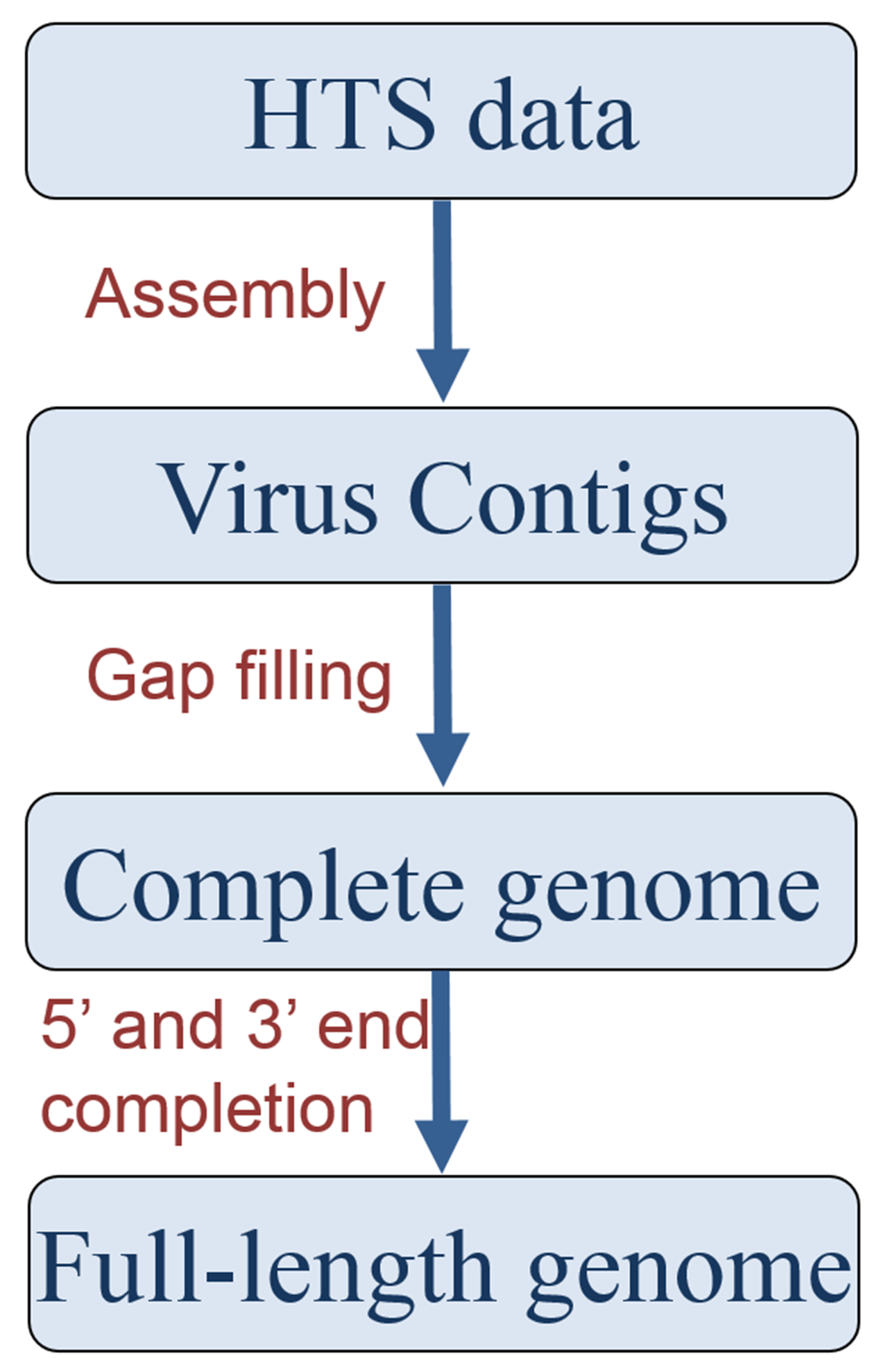


**Figure S1. A workflow to generate full-length genome sequence of an RNA virus.** “HTS” represents High throughput sequencing. “Gap flling” is usually performed using PCR amplification coupled with Sanger sequencing. 5' and 3' end completion is performed using 5' and 3' end small RNAs.

### **1.2 Collection of ticks**

Table S1. Collection of ticks

| **Species** | **Place (China)** | **Host** | **Number** | **Stage** |
| --- | --- | --- | --- | --- |
| *Amblyomma testudinarium* | Xishuangbanna,Yuannan | Buffalo | 5 | Adult |
| *Dermacentor nuttalli* | Yili,Xinjiang | Cattle | 100 | Adult |
| *D. niveus* | Yili,Xinjiang | Cattle | 100 | Adult |
| *D. silvarum* | Qingyang,Gansu | Cattle | 100 | Adult |

### **1.3 Cleaning and quality control of sRNA data**

The software Fastq_clean was used for sRNA data cleaning and quality control with the following parameter setting.

distance=2, The largest edit distance can be allowed between a adapter and reads;

max_N=0, Only report reads containing no more than given number of 'N' after trimming;

min_Len=15, Only report reads not shorter than given Length after trimming;

CPU=8, Number of processors to use;

max_dist=1, Maximum edit distance;

max_open=1, Maximum number of gap opens;

max_extension=1, Maximum number of gap extensions;

len_seed=15, Take the first INT subsequence as seed for the BWA alignment;

dist_seed=1, Maximum edit distance in the seed.

### **1.4 Kits and procedures**

RNA extraction was performed using TRIzol Reagent (Thermo Scientific, USA) following the procedure provided by the company.

The sRNA-seq library construction and sequencing were performed by the Novogene (CN) Company Limited.

Using specific primers (**Table S2**), PCR amplification coupled with Sanger sequencing was used to fill the gaps between contigs and to confirm the assembly results.

Table S2. Primers for PCR amplification coupled with Sanger sequencing

| **ID** | **Target** | **5' start** | **3' start** | **Forward / Reverse** |
| --- | --- | --- | --- | --- |
| Sanger1 | RNA1 | 8 | 1167 | AGAGGCCGCCCTTTACTGCA  GCGATCTTGTAACCGAGCTCT |
| Sanger2 | RNA1 | 1063 | 2386 | CCACTCACACCGCTGAACATG  AGTCTCATTGCCGTACTTCACC |
| Sanger3 | RNA1 | 2301 | 2952 | GCAGGAATCTGAGATCATAGTGG  TGTTGTGTTCATGTACGGTGGC |
| Sanger4 | RNA2 | 29 | 825 | CAAGTGCATACATCGACAACGA  TGGTGTAGATGGAGTACCTCCA |
| Sanger5 | RNA2 | 755 | 1697 | CTCTCCACATATGAGTACAGCT  TAGGACGATGACAAAGACTCGG |
| Sanger6 | RNA2 | 1588 | 2649 | GGTGGATCTAGTGGTCAACAAC  ATGCGGCCATAACCGGTTTC |
| Sanger7 | RNA3 | 103 | 918 | TGTTGAGAACTTGACATGGC  CTGGCTGTTCTCGATCTCAGTG |
| Sanger8 | RNA3 | 735 | 1609 | GGACAGAGCAGTATGGATTGAG  GGTGTAGACGTGCTCCTCGATA |
| Sanger9 | RNA3 | 1468 | 2657 | GCACTGGTACATCAGGCTGTG  AGCCGCAACCTAGTCATTGCT |
| Sanger10 | RNA4 | 23 | 1141 | GTTGCAAGTGCCATAGCTCG  TCGAGATACTGTCGTATCAGGC |
| Sanger11 | RNA4 | 1031 | 2137 | AAGGCTAACATCATGGGAGAGA  TAGGTCCTGCTCCACAGATG |
| Sanger12 | RNA4 | 1949 | 2685 | ACAGAGCTCTTCGGGGATGT  TCCATGCTCCGATCCGGAGA |

“ID” is unique for each Sanger sequence in the present study. RNA1, RNA2, RNA3 and RNA4 of the MGTV strain Yunnan2016 have been submitted to the NCBI GenBank database under the accession numbers MT080097, MT080098, MT080099 and MT080100, respectively.

The cDNA was performed using Thermo Scientific RevertAid First Strand cDNA Synthesis Kit K1621 (Thermo Scientific, USA). PCR amplification was performed using LA Taq (TaKaRa, Japan). Each sample mixed with PCR reagent (**Table S3**) was incubated at 94 °C for 30 s, followed by 35 PCR cycles (10 s at 98 °C, 68 m at 68 °C, and 30 s at 72 °C for each cycle).

Table S3. PCR reagent for each sample

| TaKaRa LA Taq（5 U/μL） | 12.5 µL |
| --- | --- |
| dNTP Mixture（2.5 mM each） | 1.0 µL |
| Template | 1 µg |
| primer F (10pM/µL) | 1 µL |
| primer R (10pM/µL) | 1 µL |
| RNase/DNase Free Water | - |
| Total | 50 µL |

### **1.5 MGTV, JMTV, KITV and GXTV complete genomes**

Table S4. 17 complete genomes for further analysis

| **Strain name** | **RNA1** | **RNA1** | **RNA3** | **RNA4** |
| --- | --- | --- | --- | --- |
| China2010 | KJ001579.1 | KJ001580.1 | KJ001581.1 | KJ001582.1 |
| Laos | MN095527.1 | MN095528.1 | MN095529.1 | MN095530.1 |
| France* | MN095523.1 | MN095524.1 | MN095525.1 | MN095526.1 |
| TT2017-2* | MN025516.1 | MN025517.1 | MN025518.1 | MN025519.1 |
| TT2017-1* | MN025512.1 | MN025513.1 | MN025514.1 | MN025515.1 |
| Guinea2017* | MK673133.1 | MK673134.1 | MK673135.1 | MK673136.1 |
| Xinjiang2016* | MK174251.1 | MK174244.1 | MK174230.1 | MK174237.1 |
| China2017 | MH814977.1 | MH814978.1 | MH814979.1 | MH814980.1 |
| Brazil2015* | MH155907.1 | MH155905.1 | MH155906.1 | MH155908.1 |
| Brazil2016-2 | MH155896.1 | MH155894.1 | MH155895.1 | MH155897.1 |
| Brazil2016* | MH155892.1 | MH155890.1 | MH155891.1 | MH155893.1 |
| Kosovo2015 | MH133321.1 | MH133323.1 | MH133322.1 | MH133324.1 |
| Kosovo2014 | MH133317.1 | MH133319.1 | MH133318.1 | MH133320.1 |
| Kosovo2013* | MH133313.1 | MH133315.1 | MH133314.1 | MH133316.1 |
| China2016 | MG703253.1 | MG703254.1 | MG703252.1 | MG703255.1 |
| Brazil2011 | JX390986.2 | KY523073.1 | JX390985.2 | KY523074.1 |
| Yunnan2016* | MT080097.1 | MT080098.1 | MT080099.1 | MT080100.1 |

In total, 17 MGTV, JMTV, KITV and GXTV genomes were used for the further analysis. Five phylogenetic trees from the CDS 1, 2, 3 and 4, as well as the combined CDSs, were built using nine non-redundant genome sequences (marked by *).
